# Induction of Laccase, Lignin Peroxidase and Manganese Peroxidase Activities in White-Rot Fungi Using Copper Complexes

**DOI:** 10.3390/molecules21111553

**Published:** 2016-11-17

**Authors:** Martina Vrsanska, Stanislava Voberkova, Vratislav Langer, Dagmar Palovcikova, Amitava Moulick, Vojtech Adam, Pavel Kopel

**Affiliations:** 1 Department of Chemistry and Biochemistry, Mendel University in Brno, Zemedelska 1, CZ-613 00 Brno, Czech Republic; mxtinka@seznam.cz (M.V.); stanislava.voberkova@mendelu.cz (S.V.); amitavamoulick@gmail.com (A.M.); vojtech.adam@mendelu.cz (V.A.); 2 Central European Institute of Technology, Brno University of Technology, Purkynova 123, CZ-612 00 Brno, Czech Republic; 3 Environmental Inorganic Chemistry, Department of Chemical and Biological Engineering, Chalmers University of Technology, SE-412 96 Göteborg, Sweden; langer@chalmers.se; 4 Department of Forest Protection and Wildlife Management, Faculty of Forestry and Wood Technology, Mendel University in Brno, Zemedelska 1, CZ-613 00 Brno, Czech Republic; palovcik@mendelu.cz

**Keywords:** laccase, peroxidase, copper complex, trimesic acid, enzymatic induction

## Abstract

Ligninolytic enzymes, such as laccase, lignin peroxidase and manganese peroxidase, are biotechnologically-important enzymes. The ability of five white-rot fungal strains *Daedaleopsis confragosa*, *Fomes fomentarius*, *Trametes gibbosa*, *Trametes suaveolens* and *Trametes versicolor* to produce these enzymes has been studied. Three different copper(II) complexes have been prepared ((Him)[Cu(im)_4_(H_2_O)_2_](btc)·3H_2_O, where im = imidazole, H_3_btc = 1,3,5-benzenetricarboxylic acid, [Cu_3_(pmdien)_3_(btc)](ClO_4_)_3_·6H_2_O) and [Cu_3_(mdpta)_3_(btc)](ClO_4_)_3_·4H_2_O, where pmdien = *N*,*N*,*N*′,*N*′′,*N*′′-pentamethyl-diethylenetriamine and mdpta = *N*,*N*-bis-(3-aminopropyl)methyl- amine), and their potential application for laccase and peroxidases induction have been tested. The enzyme-inducing activities of the complexes were compared with that of copper sulfate, and it has been found that all of the complexes are suitable for the induction of laccase and peroxidase activities in white-rot fungi; however, the newly-synthesized complex M1 showed the greatest potential for the induction. With respect to the different copper inducers, this parameter seems to be important for enzyme activity, which depends also on the fungal strains.

## 1. Introduction

The species of *Basidiomycetes* are considered to be a very interesting group of fungi, including different ecological groups, such as white-rot, brown-rot and leaf-litter fungi [1]. Among them, the white-rot fungi are able to efficiently decompose lignin due to the production of ligninolytic enzymes, only. Concurrently, they produce also many other types of enzymes, such as lignin and cellulose degrading enzymes (β-glucosidase, cellobiohydrolase and β-xylosidase), which can interact with the substrate [2]. They typically secrete one or more of the principal ligninolytic enzymes, i.e., phenol oxidase laccase (Lac, E.C. 1.10.3.2) and three heme peroxidases, lignin peroxidase (LiP, E.C. 1.11.1.14), manganese-dependent peroxidase (MnP, E.C. 1.11.1.13) and versatile peroxidase (VP, E.C. 1.11.1.16), depending on the fungal species, culture types and the conditions of cultivation [3]. On the other hand, most enzymes of wood-decaying fungi are identified and isolated, but the role of inducers on individual enzymes in degradation is not described sufficiently. Just a small number of secreted enzymes has been characterized, although it is known that the fungal multicomponent enzyme systems are much more complicated, and the full spectrum of effectors involved in the degradation of material remains unknown [4].

Laccase belongs to blue copper oxidase, which contains four copper atoms per molecule in the catalytic center and catalyzes the four-electron reduction of oxygen to water [5,6]. In addition, copper has also an effect at the transcriptional level. Thus, this metal regulates mRNA levels of laccase-encoding genes in different fungi, for example *Trametes versicolor* [7], *Trametes pubescens* [8] and *Pleurotus ostreatus* [9]. To date, it is not known with certainty how copper activates the transcription of laccase genes [10]. The laccase activity in fungal cultures, however, does not necessarily correspond to the level of gene expression [11]. In addition to the chemical structure of laccase inductors, the chemical concentration also plays an important role in the regulation of gene expression in *Trametes* sp. [12]. A significant advantage of this enzyme is the ability to oxidize a huge variety of organic or inorganic compounds, including phenols (e.g., catechol, hydroquinone, 2,6-dimethoxyphenol and syringaldazine), aromatic amines and ascorbate [13]. Thus, it is not surprising that laccase is often produced in the form of numerous isoenzymes due to culture strain, conditions and the addition of inducting agents to the culture medium [9,13].

Lignin peroxidase is a glycosylated protein-containing heme. In the presence of endogenously-produced peroxide, LiP catalyzes the oxidation of aromatic non-phenolic lignin structures to give aryl-cation radicals. The oxidative lignin degradation requires the presence of veratryl alcohol, which is the substrate for LiP and also a secreted fungal metabolite. Manganese-dependent peroxidase also contains heme. It catalyzes a H_2_O_2_-dependent oxidation of Mn^2+^ to form highly reactive Mn^3+^, which subsequently oxidizes the phenolic parts of lignin to produce free radicals. The high reactivity of Mn^3+^ is stabilized by chelates (oxalate, malonate, maleate), which are secreted by fungi [14]. The synthesis and secretion of these enzymes are highly influenced by the nutrient levels (N/C ratio), culture conditions, fungal developmental state, as well as the addition of inducing agents to the culture medium [5].

The use of enzymes for many purposes, however, entails a number of limitations. The ligninolytic enzymes from white-rot fungi are secreted in small amounts only, so their use in industrial applications has been limited due to the low productivity and high cost [15]. Therefore, new and less expensive sources of enzymes are constantly being investigated. A higher enzyme activity guarantees a higher and faster transformation of the target substrate and improves the applicability and effectiveness of the enzyme-catalyzed processes [16]. From this point of view, the enzymatic induction of white-rot fungi is very important since their metabolic activity and growth are dependent on the environmental conditions. The addition of inducers, such as xylidine [17], ferulic acid, veratryl alcohol, pyrogallol and copper [9,11], has been found to increase laccase secretion. In the work of Shah et al. [2], nanoparticles of copper significantly induced the production of ligninolytic enzymes and reduced the secretion of hydrolytic enzymes (β-glucosidase, β-xylosidase and cellobiohydrolase).

Copper sulfate was efficiently used as an inducer to increase laccase production of 85% [18]. Previous studies have shown that copper is an efficient inducer of laccase when supplemented in the medium in copper sulfate form by increasing the transcription and the activity of enzyme [19]. Therefore, one may assume that some newly-synthetized molecules could be the way to induce enzyme production with higher efficiency.

1,3,5-benzenetricarboxylic acid (trimesic acid) (H_3_btc) is a tricarboxylic acid with potentially six oxygen donor atoms available for coordination to the central atoms. Many complexes with the benzenetricarboxylate bridge have been studied, and different coordinations of the carboxylate groups have beenproven [20,21,22]. The complexes with *N*-donor ligands and carboxylic acids are studied not only for their interesting magnetic and spectral properties, but also for applications as catalysts [23,24], nano-material precursors [25] and for their antibacterial, antiviral and antitumor activities [26]. On the other hand, the complexes have never been used to induce enzyme activity.

Here, we present the preparation and characterization of a new copper complex (Him)[Cu(im)_4_(H_2_O)_2_](btc)·3H_2_O (M1), where im = imidazole. Its structure was proven by single crystal X-ray analysis. The main scope of this study was to use the newly-synthesized copper complex (M1) and already known trinuclear copper complexes with the btc bridge [27] [Cu_3_(pmdien)_3_(btc)](ClO_4_)_3_·6H_2_O (M2) and [Cu_3_(mdpta)_3_(btc)](ClO_4_)_3_·4H_2_O (M3), where pmdien = *N*,*N*,*N*′,*N*′′,*N*′′-pentamethyl-diethylenetriamine and mdpta = *N*,*N*-bis-(3-aminopropyl)methylamine, and to test their ability to increase the enzyme activity of white-rot fungi as inducers.

## 2. Results and Discussion

### 2.1. Synthesis and Characterization of Complexes

**Figure 1:**
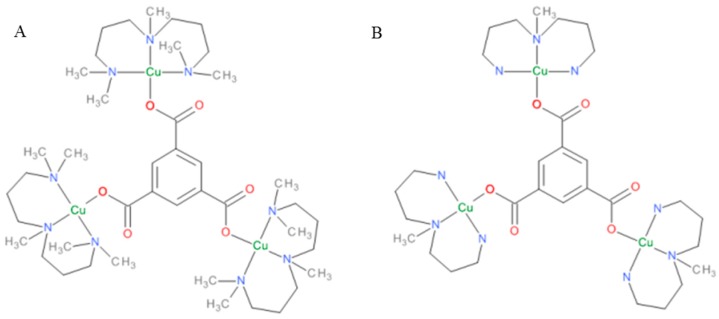
Structural formulas of the complex cations [Cu_3_(pmdien)_3_(btc)]^3+^, where pmdien *= N*,*N*,*N*′,*N*′′,*N*′′-pentamethyl-diethylenetriamine, (M2) (**A**) and [Cu_3_(mdpta)_3_(btc)]^3+^, where mdpta = *N*,*N*-bis-(3-aminopropyl)methylamine), (M3) (**B**).

The polymer precursor Cu_3_(btc)_2_·10H_2_O, was prepared as a precipitate in the reaction of copper chloride with sodium salt of 1,3,5-benzenetricarboxylic acid and used to react with imidazole. Although we expected the formation of a polynuclear complex with the btc bridge, only a mononuclear copper complex was formed, which was characterized by elemental analyses, UV/VIS and IR spectroscopies. There was only one broad band in the UV/VIS spectra typical for the Cu(II) ion at 16,600 cm^−1^ that belongs to the d-d transition. In the IR spectrum, the strong peaks at 829 and 1262 cm^−1^ belong to νN-H and νC-N vibrations of imidazole, respectively, whereas the strong vibrations at 1357 and 1609 cm^−1^ are most probably the ν_s_COO and ν_as_COO vibrations of carboxylate groups [28]. The complex M1 exhibited an effective magnetic moment 1.76 B.M., which is characteristic for one unpaired electron of the Cu(II) ion. Moreover, the structure was proven by the single crystal X-ray analyses. The complexes M2 and M3 have been characterized in the literature, and their structures are depicted in Figure 1.

### 2.2. X-ray Structure of (Him)[Cu(im)_4_(H_2_O)_2_](btc)·3H_2_O (M1)

**Figure 2:**
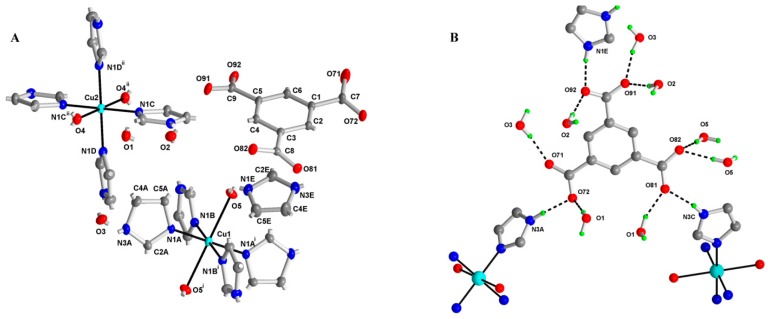
Numbering scheme for M1with atomic displacement ellipsoids drawn at the 50% probability level. Hydrogen atoms omitted for clarity (**A**). Hydrogen-bond pattern of the benzenetricarboxylic ion. The hydrogens involved in hydrogen bonds are shown only (**B**). Oxygen (red), nitrogen (dark blue), copper (light blue), hydrogen (green).

The numbering scheme of the M1 complex is shown in Figure 2A, and selected bond lengths and angles are listed in Table 1. The structure of (imidazolium) trans-bis(aqua-κO)tetrakis(1*H*-imidazole-κN^3^)copper(II) (1,3,5-benzenetricarboxylate) trihydrate (C_3_H_5_N_2_)[Cu(C_3_H_4_N_2_)_4_(H_2_O)_2_](C_9_H_3_O_6_)·3H_2_O, contains copper(II) and imidazolium cations, the 1,3,5-benzenetricarboxylate (btc^3−^) anion and three water molecules. Both Cu(II) central atoms, located on the inversion center, are coordinated by four nitrogen atoms of imidazole (im) in square planar arrangement, although coordination Number 6 and deformed octahedral arrangement can be assumed if the interaction of oxygen atoms of two water molecules is taken into account. The structure is stabilized by an extensive network of hydrogen bonds connecting the btc^3−^ anion, imidazolium, the copper cation and crystal water molecules; see Table 2 and Figure 2B. There is also observed a *π*-*π* interaction between imidazole rings N1D/C2D/N3D/C4D/C5D with centroid-centroid (symmetry code: −x, 1 − y, 1 − z) distance of 3.6773 (13) Å and slippage of 1.603 Å. Another weak interaction is of C-H…*π* type: C7-O71…centroid (N1E/C2E/N3E/C4E/C5E) (symmetry code: 1 + x, y, z) is 3.472 (2) Å with an angle of 93.94 (14)°.

### 2.3. Comparative Evaluation of White-Rot Fungal Strains

A total of 35 fungal strains isolated from a Czech forest were evaluated in terms of their ability to produce enzyme laccase with a high enzyme activity. Among the white-rot fungi examined, *Fomes fomentarius*, *Trametes suaveolens*, *Daedaleopsis confragosa*, *Trametes versicolor* and *Trametes gibbosa* exhibited the highest enzyme activity during 32 days on cultivation medium without induction [29].

**Figure 3:**
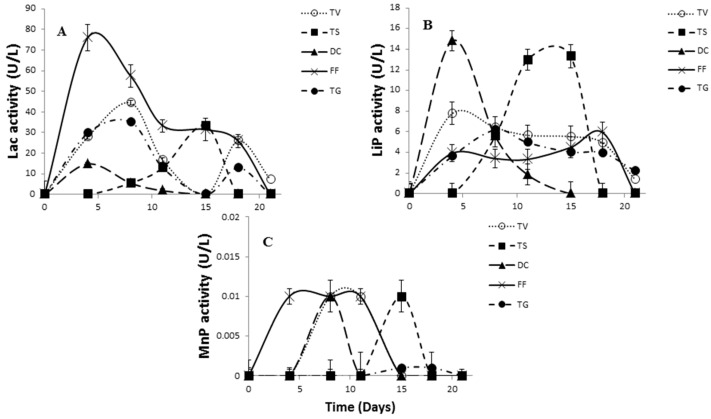
Evaluation of laccase (Lac) (**A**); lignin peroxidase (LiP) (**B**) and manganese-dependent peroxidase (MnP) (**C**) activities of tested white-rot fungi without induction. Standard deviation lines represent the variation of mean activity value obtained from three replicate cultivations. The strains and abbreviations: *Trametes versicolor* (TV); *Trametes suaveolens* (TS); *Daedaleopsis confragosa* (DC); *Fomes fomentarius* (FF); *Trametes gibbosa* (TG).

The literature data reporting laccase activity of white-rot fungi show that *Trametes* sp. is a significant producer of ligninolytic enzymes [29], which can be highly substrate specific [30]. Therefore, many studies with these strains have been extensively conducted [31,32]. The results of our study indicated that *Fomes fomentarius* seems to be one of the best producers of laccase with the highest activity (nearly 80 U/L) after four days of incubation (Figure 3A). The results agree with the study of Songulashvili et al. [33], where *Fomes fomentarius* was observed as the best producer of laccase. Opposite results were published in the work of Rodrigues et al. [34], where *Trametes versicolor* was the better laccase producer in comparison with *Fomes fomentarius*. *Trametes gibbosa* exhibited a similar trend in Lac production as *Trametes versicolor* (Figure 3A). In contrast to the above fungal strains, the laccase activity of *Daedaleopsis confragosa* and *Trametes suaveolens* was insignificant.

The ability of *Fomes fomentarius, Trametes versicolor* and *Trametes gibbosa* for ligninolytic peroxidases production is well established [11]. To our knowledge, there is no information on ligninolytic peroxidase secretion by the white-rot fungus *Daedaleopsis confragosa* and *Trametes suaveolens.* Surprisingly, *Daedaleopsis confragosa* and *Trametes suaveolens* exhibited the highest activity of peroxidase enzymes (lignin LiP and manganese MnP) reaching values greater than 14 U/L for LiP (Figure 3B). However, MnP showed very low activity for all fungal strains (Figure 3C), which is in accordance with the outcome of manganese peroxidase secretion by *Fomes* and *Trametes* strains [14]. In the study by Jarvinen et al. [35], who tested the MnP activity of white-rot fungi, it was observed that the initial MnP enzyme amounts were very low, and they concluded that there were significant differences in the activity of MnP enzymes from different white-rot fungi due to the secretion of many isoforms. On the other hand, Arantes and Milagres [36] reported the production of MnP by white-rot fungi, and this enzyme was the main enzyme produced by all fungi, especially by *Trametes versicolor*.

### 2.4. Effect of Inducers Concentration on Lac, LiP and MnP Activities

Heavy metals in general are potent inhibitors of enzymatic reactions, and from this point of view, their concentration plays very important roles. Usually, the metals start to be toxic for the fungus in a concentration only a few times greater than that required [37]. Copper can cause oxidative damage of proteins by the induction of oxidative stress, but concurrently serve as a cofactor in the catalytic center of laccase [1]. Collins and Dobson [7] reported that the laccase expression of the white-rot fungus *Trametes versicolor* is regulated by copper at the gene transcription level. In general, the cultures enriched with copper show significant laccase activity. Copper ions induce also manganese peroxidase activity, but there are very few studies concerning the effect of copper on lignin peroxidase [38]. In our work, three types of copper complexes (M1, M2, M3) together with copper sulfate (Cu) in four different concentrations (0.1, 0.3, 0.5 and 1.0 mM) were used for examining the Lac, MnP and LiP production (Table 3).

For all tested white-rot fungi, the increase in Lac activity was found to be proportional to the amount of copper sulfate (Cu) added, and the best concentration of copper seemed to be 1 mM (Table 3). However, if the copper complexes were used, comparable Lac activity was observed, while a lower concentration of the inductor was added. This is in contrast to the previous studies, where the species-specific increase in laccase production dependent on copper concentration ranging from 2 µM to 2 mM was observed [6,8,9,39]. The LiP activity varied depending on the type of inductor and strain used. However, the copper complexes had similar or better activity than copper sulfate (Table 3). On the other hand, no significant changes on the MnP activity were observed after the addition of copper inducers, as anticipated, because this activity was negligible. The same results were observed in the study of Shah et al. [2], where aggregated copper nanoparticles showed no influence on the production of Mn-peroxidase.

Considering all of these data, it can be concluded that not only copper ions, but also the type of copper compounds play an important role, especially in Lac and LiP activities.

### 2.5. The Effect of Inducers on Lac, LiP and MnP Activities

Although the positive effect of copper addition (in the form of copper sulfate) on laccase activity has been previously reported [38], the reason why copper effectively stimulates laccase synthesis is at present not clearly understood, and concurrently, the effect of the structure of the copper inducer on the enzyme activity has not been studied yet. Numerous studies have been carried out for the comparison of the type of inducer and the concentration, as well as the composition of the culture medium on the laccase and peroxidase activity [11]. However, there is no information of the enzyme-inducing effect of the copper complexes on the white-rot fungi.

**Figure 4:**
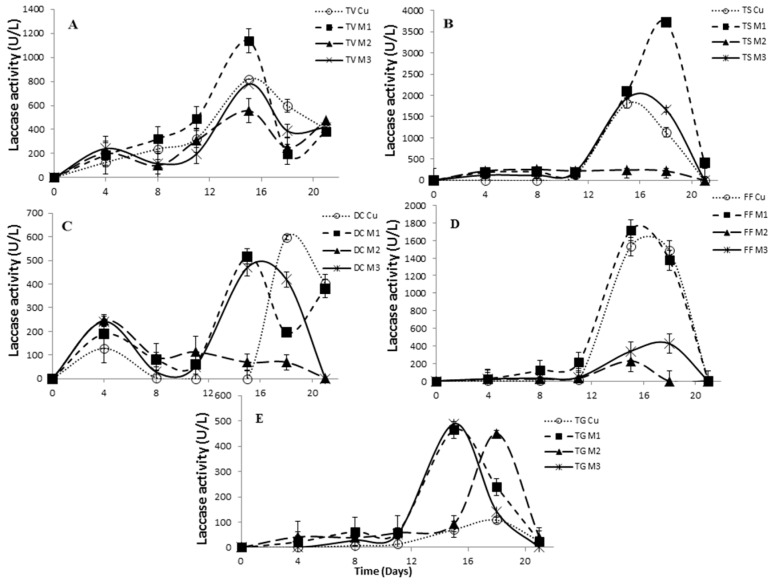
Evaluation of laccase activity of TV, *Trametes versicolor* (**A**); TS, *Trametes suaveolens* (**B**); DC, *Daedaleopsis confragosa* (**C**); FF, *Fomes fomentarius* (**D**); and TG, *Trametes gibbosa* (**E**); with a 0.5 mM concentration of inducers. Standard deviation lines represent the variation of the mean activity value obtained from three replicate cultivations.

In our study, three copper complexes differing in their structure were tested as new inducers of Lac, LiP and MnP activity, and the results were compared with traditionally-used copper sulfate. All of the inducers tested using *Trametes versicolor* effectively stimulated the Lac activity. There was observed significant differences between copper sulfate and copper complexes, indicating the effect of inductor structure on the Lac activity. All inducers showed a similar trend, when the laccase activity increased after 15 days of cultivation. The highest Lac activity of *Trametes versicolor* was examined using the newly-synthesized complex M1 (Figure 4A), where imidazole was included in the complex structure (Figure 2A).

The same results were determined in the case of *Trametes suaveolens*, where the laccase activity induced by the complex M1 showed a 118-fold increase (around 2000 to 3700 U/L) in comparison with the sample without copper induction after 15 days of incubation. However, the addition of the complex M2 rapidly decreased the laccase activity. Copper sulfate demonstrated a similar trend as complex M3, where the highest Lac activity was observed after 15 days (Figure 4B).

*Daedaleopsis confragosa* exhibited the highest Lac activity due to the addition of copper sulfate (reaching values of 600 U/L after 18 days), although the complexes M1 and M3 showed comparable activity also after 15 days (Figure 4C). *Fomes fomentarius* showed the highest laccase activity 1700 U/L) after 15 days using the complex M1, and a similar trend had also been observed for copper sulfate (Figure 4D). A stimulatory effect of the complexes on the enzyme production was observed also for *Trametes gibbosa* strain, where the Lac activity was 3.2-fold higher than the activity found for copper sulfate after 15 days (Figure 4E).

**Figure 5:**
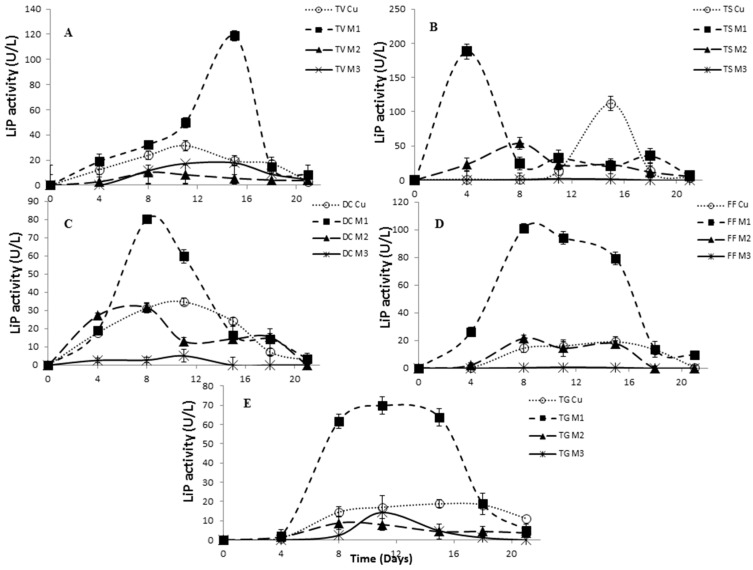
Evaluation of lignin peroxidase activity of TV, *Trametes versicolor* (**A**); TS, *Trametes suaveolens* (**B**); DC, *Daedaleopsis confragosa* (**C**); FF, *Fomes fomentarius* (**D**); and TG, *Trametes gibbosa* (**E**), with a 0.5 mM concentration of inducers. Standard deviation lines represent the variation of the mean activity value obtained from three replicate cultivations.

As regards LiP activity, the most important inducer seems to be the complex M1; however, the maximum activity depends on the cultivation day. For example, the highest level of LiP activity for *Trametes versicolor* was found after 15 days of cultivation (Figure 5A). Very high increases (nearly 20-fold) of LiP activity were observed for *Trametes suaveolens* in the presence of the newly-synthesized complex M1 after four days of cultivation (Figure 5B). The addition of the same complex M1 significantly stimulated the LiP production also for other fungal strains *Daedaleopsis confragosa*, *Fomes fomentarius* and *Trametes gibbosa*, reaching the maximum between 8 and 15 days of cultivation (Figure 5C–E).

**Figure 6:**
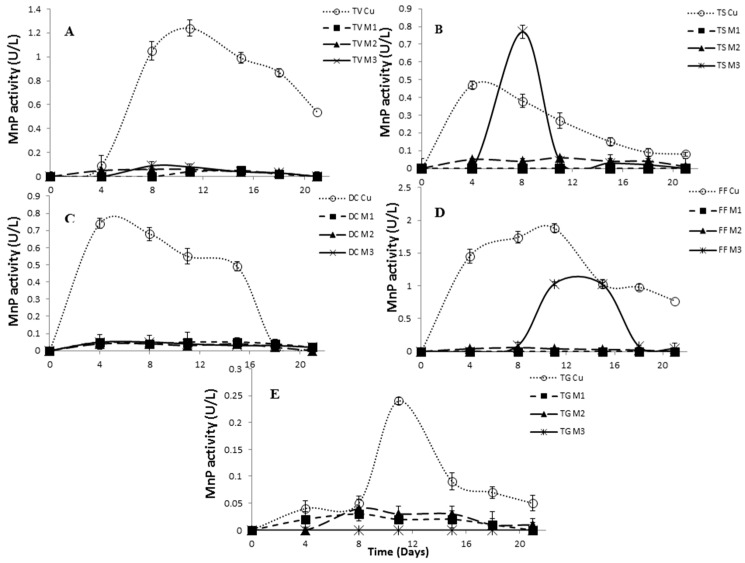
Evaluation of manganese-dependent peroxidase activity of TV, *Trametes versicolor* (**A**); TS, *Trametes suaveolens* (**B**); DC, *Daedaleopsis confragosa* (**C**); FF, *Fomes fomentarius* (**D**); and TG, *Trametes gibbosa* (**E**), with a 0.5 mM concentration of inducers. Standard deviation lines represent the variation of mean activity value obtained from three replicate cultivations.

MnP activity was negligible for all three copper complexes in comparison with copper sulfate for all tested strains (Figure 6A–E). It was assumed that MnP activity would be low, because manganese is the most important metal inducer for white-rot fungi as has been reported by many authors [40,41].

All of these observations indicated that the induction effect was dependent not only on the fungal strain, but also on the structure of the copper compound. The addition of the inducers, especially in our case the newly-synthesized copper complex M1, had a major effect on the secretion of laccase and lignin peroxidase by *Trametes suaveolens*. One explanation for this observation is connected with the similarity of the copper complex (third metal-binding pocket with imidazole in the active center) with the molecular structure of laccase. Loera Corral et al. [42] reported that the increase in laccase production is the result of the analogy of the inducer structure with lignin or other chemical agents. Lower or comparable laccase activity of M3 with Cu can be explained by the availability of coordination places around the central copper atom (coordination Number 4), whereas very low laccase activity in most cases of M2 can be caused by the presence of methyl groups on the pmdien ligand. The methyl groups are sterically demanding, and for example, in nickel complexes with pmdien ligand, coordination Number 4 instead of preferred octahedral arrangement was proven [43,44], although sometimes, water can be present and increases the coordination number to 6 [45]. This is probably a reason why apical positions are protected for the interactions with electron pairs of the functional groups.

Furthermore, the biotransformation of the copper complex M1 with imidazole units by the extracellular enzymes Lac or LiP can lead to the formation of products that enhance enzyme activity. Consequently, further studies revealing the detailed mechanism of induction with respect to the structure of the produced enzymes and potential transformation of inducers are necessary.

## 3. Materials and Methods

### 3.1. Chemicals and Instruments

The chemicals and solvents were supplied by Aldrich (St. Louis, MO, USA) and used as received. The C, H and N analyses were carried out on an EA 1108 instrument (Fisons Instruments, Rodano, Italy). The IR spectra (400 to 4000 cm^−1^) were recorded on an FTIR spectrometer (Bruker Tensor 27, Billerica, MA, USA) using KBr pellet. The absorption spectra were obtained using a Specord 210 UV/VIS spectrometer (Analytik Jena AG, Jena, Germany).

The crystallographic data for M1 have been deposited with the Cambridge Crystallographic Data Centre as Supplementary Publication No. 1439845. The copies of the data can be obtained, free of charge, on application to CCDC, 12 Union Road, Cambridge, CB2 1EZ, U.K. (Fax: +44-(0)1223-336033 or e-mail: deposit@ccdc.cam.ac.uk).

### 3.2. Preparation of the Complexes

#### 3.2.1. Cu_3_(btc)_2_·10H_2_O

CuCl_2_·2H_2_O (6.7 g, 40 mM) was added to an aqueous solution of Na_3_btc (250 mL) prepared by the reaction of H_3_btc (5.5 g, 26 mM) with NaOH (3.2 g, 80 mM). The reaction mixture was refluxed for 4 h. After cooling, the blue precipitate was filtered off, washed with water and dried in air. Yield: 72%. Anal. (%) Calc.: C, 27.54; H, 3.34; Cu, 24.28. Found: C, 27.83; H, 3.31; Cu, 24.31.

#### 3.2.2. (Him)[Cu(im)_4_(H_2_O)_2_](btc)·3H_2_O: Complex M1

Imidazole (im) (0.41 g, 6 mM) dissolved in EtOH (25 mL) was added to the suspension of Cu_3_(btc)_2_·10H_2_O (0.39 g, 0.5 mM) in water (50 mL). The mixture was heated to boiling and stirred for 2 h. The solution was filtered and left for crystallization. Dark blue crystals, suitable for X-ray analysis, were obtained after a week, separated by filtration, washed with cold water and dried in air. Yield: 12%. Anal. (%) Calc.: C, 41.05; H, 4.88; N, 19.95; Cu, 9.05.Found: C, 41.12; H, 4.53; N, 19.48; Cu, 10.12. UV/VIS (×10^3^ cm^−1^): λ_max_ 16.6; 47.7. IR (cm^−1^): 619, 658, 711, 829 (νN-H), 1070, 1147, 1180, 1262 (νC-N), 1357 (ν_s_COO), 1427, 1565, 1609 (ν_as_COO), 1662, 2853, 2927, 3137, 3415. µ_eff_/B.M. = 1.76.

#### 3.2.3. [Cu_3_(pmdien)_3_(btc)](ClO_4_)_3_·6H_2_O: Complex M2

The complex was prepared according to Kopel et al. [27]. Briefly, pmdien (0.21 mL, 1 mM) was mixed with a solution of Cu(ClO_4_)_2_·6H_2_O (0.37 g, 1 mM) in water (25 mL). A blue precipitate obtained after Na_3_btc solution addition (0.1 M, 3.3 mL) was filtered off, washed with EtOH and dried.

#### 3.2.4. [Cu_3_(mdpta)_3_(btc)](ClO_4_)_3_·4H_2_O: Complex M3

The complex was prepared according to a published method [27], whereas *N*,*N*-bis-(3-aminopropyl)methylamine (mdpta) was used instead of pmdien, only.

### 3.3. X-ray Crystallography

X-ray data of M1 were collected on a SMART CCD diffractometer (Siemens, Madison, WI, USA) with Mo-Kα radiation (λ = 0.71073 Å). The crystal was cooled to 173(2) K by a flow of nitrogen gas using the LT-2A device. Preliminary orientation matrices were obtained from the first frames using SMART (Area Detector Control and Integration Software, version 5.63, Bruker AXS Inc., Madison, WI, USA, 2003). Final cell parameters were obtained by refinement of the positions of reflections with I > 10σ (I) after integration of all of the frames using SAINT software (Area Detector Control and Integration Software, Bruker AXS Inc., version 6.45, Madison, WI, USA). The data were empirically corrected for absorption and other effects using the SADABS program (Program for Empirical Absorption Correction for Area Detectors (Version 2.10, University of Gottingen, Gottingen, Germany), Sheldrick G.M., University of Gottingen, Gottingen, Germany, 2003). The structure was solved by direct methods and refined by full-matrix least squares on all |F^2^| data using SHELXTL software (version 2.10, University of Gottingen, Gottingen, Germany) [46]. Reflections 0 1 0, 0 −1 1 and 0 0 1 were omitted as they were in the shadow of the primary beam stop. Hydrogen atoms were refined isotropically. Those attached to N were constrained to an ideal geometry, riding on the pivot atoms (N-H = 0.88 Å), while water hydrogens were refined freely. X-ray data of M1: the important crystallographic parameters are as follows: C_12_H_20_N_8_O_2_Cu·C_9_H_3_O_6_·C_3_H_5_N_2_·3(H_2_O), wavelength 0.71073 Å, triclinic, space group Pī, *a* = 8.4057(4), *b* = 14.0214(6), *c* = 14.5608(6) Å, α = 114.701(1)°, β = 96.927(1)°, γ = 91.115(1)°, volume 1543.08(12) Å^3^, Z = 2, crystal size 0.72 mm × 0.69 mm × 0.22 mm, index ranges –11 ≤ *h* ≤ 11, −20 ≤ *k* ≤ 20, −20 ≤ *l* ≤ 20, reflections collected/independent 25,634/9391 (*R*_int_ = 0.0458, data/restraints/parameters 9391/0/482, goodness-of fit on *F*^2^ = 1.032, final *R*_1_ (I > 2σ(I) data) = 0.0400, *wR*_2_ = 0.0901, final *R*_1_ (all data) = 0.0706, *wR*_2_ = 0.1001. The largest peak and hole on the final difference map were 0.428 and −0.702 e·Å^−3^.

### 3.4. Microorganisms, Substrates, Inocula

Five fungal strains (*Daedaleopsis confragosa* (DC), *Fomes fomentarius* (FF), *Trametes gibbosa* (TG), *Trametes suaveolens* (TS) and *Trametes versicolor* (TV)) obtained from the Culture Collection of the Faculty of Forestry and Wood Technology of the Mendel University in Brno (Brno, Czech Republic) were used in this study. All of the strains were microscopically identified and preserved on potato dextrose agar (PDA) at 4 °C and periodically sub-cultured to maintain their viability.

The cultures were grown on PDA for 10 days at 22 °C. After this, three 1 × 1 cm^2^ plugs were cut and added into Erlenmeyer flasks containing 40 mL of the potato dextrose broth (PDB). Three different copper complexes and CuSO_4_·5H_2_O were used in four different concentrations (0.1, 0.5, 1 and 5 mM). A flask for each of the strains without any inducer was used as the control. The flasks were incubated in a shaker (150 rpm, 28 °C) and the samples were measured every 4 days, in total 24 days. The supernatant was separated from the mycelium by centrifugation (10,000 rpm, 4 °C, 5 min), and the enzyme activities were determined.

### 3.5. Enzyme Activity Assay

#### 3.5.1. Laccase Activity

The laccase activity was measured at 415 nm by detecting the oxidation of 10 mM ABTS (2,2′-azino-bis(3-ethylbenzothiazoline-6-sulfonate, Sigma Aldrich, St. Louis, MO, USA, ε = 36,000 M^−1^∙cm^−1^) at pH 4.5 in 0.1 M sodium acetate buffer [32].

#### 3.5.2. Lignin Peroxidase Activity

The LiP activity was determined by the formation of veratraldehyde from 25 mM veratryl alcohol (VA, Sigma Aldrich, ε = 9300 M^−1^∙cm^−1^) in 100 mM tartrate buffer (pH 3.0). The reaction started by the addition of hydrogen peroxide (54 mM), and the appearance of veratraldehyde was determined at 310 nm.

#### 3.5.3. Manganese-Dependent Peroxidase Activity

The MnP activity was measured by 20 mM dimethylsulfoxide (DMP, Sigma Aldrich, ε = 49,600 M^−1^∙cm^−1^) oxidation method in 65.8 mM malonate buffer (pH 4.5) at 469 nm as described by Martinez et al. [46]. The enzyme activities were determined spectrophotometrically using a UV/VIS Lambda 25 Spectrophotometer (Perkin-Elmer, Shelton, CT, USA). One unit of the enzyme activity was defined as 1 μM of substrate oxidized per minute under the assay conditions. The enzyme activity assay was always performed in triplicate. The results were analyzed using the mean and standard deviation.

## 4. Conclusions

In this study, the enzyme activities have been found to be dependent on the concentration of the copper inducers and the strains of fungus. The copper complexes were successfully used as potential inducers of ligninolytic enzymes, and concurrently, the copper complex M1 exhibited the most pronounced stimulatory effect. Nevertheless, further investigations to reveal the structure of the produced enzyme with respect to the inducer structure will contribute to understanding the mechanism of action of these enzymes.

## Figures and Tables

**Table 1:** Selected bond lengths (Å) and angles (°) for M1.

Cu(1)-N(1B)	2.0099 (15)	Cu(2)-N(1D)	2.0156 (15)
Cu(1)-N(1B) ^i^	2.0099 (15)	Cu(2)-N(1D) ^ii^	2.0156 (15)
Cu(1)-N(1A) ^i^	2.0219 (14)	Cu(2)-N(1C) ^ii^	2.0219 (14)
Cu(1)-N(1A)	2.0220 (14)	Cu(2)-N(1C)	2.0220 (14)
N(1B)-Cu(1)-N(1B) ^i^	180.0	N(1D)-Cu(2)-N(1D) ^ii^	179.999 (2)
N(1B)-Cu(1)-N(1A) ^i^	90.23 (6)	N(1D)-Cu(2)-N(1C) ^ii^	90.65 (6)
N(1B)i-Cu(1)-N(1A) ^i^	89.77 (6)	N(1D) ^ii^-Cu(2)-N(1C) ^ii^	89.35 (6)
N(1B)-Cu(1)-N(1A)	89.77 (6)	N(1D)-Cu(2)-N(1C)	89.35 (6)
N(1B)i-Cu(1)-N(1A)	90.23 (6)	N(1D) ^ii^-Cu(2)-N(1C)	90.65 (6)
N(1A)i-Cu(1)-N(1A)	180.0	N(1C) ^ii^-Cu(2)-N(1C)	180.0

Symmetry transformations used to generate equivalent atoms: ^i^: −x, − y + 1, −z; ^ii^: − x + 1, − y + 1, − z + 1.

**Table 2:** Hydrogen bonds for M1 (Å) and (°).

D-H...A	d(D-H)	d(H...A)	d(D...A)	<(DHA)
O(1)-H(11)...O(72) ^iii^	0.80 (3)	1.91 (3)	2.712 (2)	174 (3)
O(1)-H(12)...O(81) ^iv^	0.86 (3)	1.90 (3)	2.7145 (19)	159 (3)
O(2)-H(21)...O(91) ^i^	0.80 (3)	1.95 (3)	2.734 (2)	169 (3)
O(2)-H(22)...O(92) ^v^	0.83 (3)	1.90 (3)	2.726 (2)	170 (3)
O(3)-H(31)...O(91) ^vi^	0.87 (3)	1.84 (3)	2.699 (2)	167 (2)
O(3)-H(32)...O(71) ^v^	0.78 (3)	1.85 (3)	2.616 (2)	170 (3)
O(4)-H(41)...O(2) ^ii^	0.80 (3)	2.04 (3)	2.825 (2)	166 (2)
O(4)-H(42)...O(3) ^vii^	0.86 (3)	1.92 (3)	2.771 (2)	173 (2)
O(5)-H(51)...O(82) ^i^	0.80 (2)	1.95 (3)	2.7472 (19)	177 (2)
O(5)-H(52)...O(82) ^iv^	0.79 (3)	2.04 (3)	2.826 (2)	174 (2)
N(3A)-H(3A)...O(72) ^viii^	0.88	1.86	2.7312 (19)	170.3
N(3B)-H(3B)...O(2) ^i^	0.88	1.99	2.853 (2)	168.2
N(3C)-H(3C)...O(81) ^iv^	0.88	1.96	2.813 (2)	162.6
N(3D)-H(3D)...O(3) ^i^	0.88	1.95	2.792 (2)	159.9
N(1E)-H(1E)...O(92) ^v^	0.88	1.9	2.773 (2)	172.2
N(3E)-H(3E)...O(1) ^ix^	0.88	1.81	2.684 (2)	174.1

Symmetry transformations used to generate equivalent atoms: ^i^: −x,− y + 1, −z; ^ii^: − x + 1, − y + 1, −z + 1; ^iii^: x, y−1, z; ^iv^: − x + 1, − y + 1, − z; ^v^: − x + 1, − y + 2, − z + 1; ^vi^: x − 1, y , z; ^vii^: −x, − y + 1, − z + 1; ^viii^: x − 1, y − 1, z; ^ix^: x, y + 1, z.

**Table 3:** Enzyme activities of Lac, LiP and MnP using different concentrations (0.1, 0.3, 0.5 and 1.0 mM) of copper sulfate and complexes M1, M2 and M3 for the tested fungal strains *Trametes versicolor* (TV); *Trametes suaveolens* (TS); *Daedaleopsis confragosa* (DC); *Fomes fomentarius* (FF); *Trametes gibbosa* (TG) after 15 days of cultivation.

0.1 mM	Lac (U/L)	LiP (U/L)	MnP (U/L)	0.3 mM	Lac (U/L)	LiP (U/L)	MnP (U/L)	0.5 mM	Lac (U/L)	LiP (U/L)	MnP (U/L)	1.0 mM	Lac (U/L)	LiP (U/L)	MnP (U/L)
		**TV**				**TV**				**TV**				**TV**	
**Cu**	583.30	9.44	0.54	**Cu**	708.30	37.40	0.15	**Cu**	819.40	31.50	1.20	**Cu**	1291.70	1.81	0.09
**M1**	833.30	333.30	0.02	**M1**	777.80	43.90	0.14	**M1**	1138.90	118.80	0.05	**M1**	533.30	1.76	0.06
**M2**	1194.40	50.20	0.05	**M2**	1472.20	26.70	0.05	**M2**	555.60	11.60	0.06	**M2**	344.60	0.50	0.48
**M3**	1305.60	135.40	0.06	**M3**	1416.70	25.30	0.03	**M3**	777.80	17.80	0.09	**M3**	777.80	11.70	0.05
		**TS**				**TS**				**TS**				**TS**	
**Cu**	987.50	17.50	0.05	**Cu**	444.40	17.80	0.09	**Cu**	1813.50	112.50	0.47	**Cu**	2108.30	1.90	0.08
**M1**	186.10	0	0	**M1**	175.00	15.30	0.02	**M1**	3725.20	187.80	0	**M1**	1234.70	1.60	0
**M2**	152.80	52.80	0.03	**M2**	206.90	15.60	0.05	**M2**	253.90	53.90	0.06	**M2**	11.70	0.31	0.31
**M3**	627.80	44.40	0.04	**M3**	306.80	26.10	0	**M3**	1941.70	2.04	0.77	**M3**	5.10	0.55	0.05
		**DC**				**DC**				**DC**				**DC**	
**Cu**	569.40	69.40	0.25	**Cu**	569.40	16.30	0.24	**Cu**	597.20	34.80	0.74	**Cu**	750.00	0.98	0.38
**M1**	272.20	42.20	0.01	**M1**	500.60	15.60	0	**M1**	516.70	80.30	0.05	**M1**	413.90	1.70	0
**M2**	83.30	16.40	0	**M2**	142.30	8.90	0.05	**M2**	241.20	31.60	0.04	**M2**	175.40	0.65	0.27
**M3**	84.20	21.40	0.05	**M3**	469.40	460.10	0	**M3**	434.70	5.10	0.05	**M3**	409.40	0.33	0
		**FF**				**FF**				**FF**				**FF**	
**Cu**	1022.20	13.10	0.24	**Cu**	1133.30	14.40	0.05	**Cu**	1529.20	17.70	0.05	**Cu**	1551.40	1.50	0.69
**M1**	433.30	54.30	0.03	**M1**	1980.60	18.10	0.06	**M1**	1719.40	101.20	0.06	**M1**	715.30	2.00	0.07
**M2**	227.80	13.30	0.07	**M2**	311.10	31.40	0.05	**M2**	227.80	21.60	0.06	**M2**	325.00	1.78	1.70
**M3**	286.10	27.40	0.08	**M3**	513.90	19.90	0.04	**M3**	425.00	0.74	1.03	**M3**	425.00	0.40	0.08
		**TG**				**TG**				**TG**				**TG**	
**Cu**	100.00	16.70	0.02	**Cu**	106.90	18.50	0.04	**Cu**	108.30	18.80	0.24	**Cu**	156.90	0.30	0.25
**M1**	80.60	70.40	0.01	**M1**	91.70	23.70	0	**M1**	463.90	69.80	0.03	**M1**	136.10	0.60	0
**M2**	350.00	38.90	0	**M2**	169.40	31.20	0.05	**M2**	450.40	8.00	0.04	**M2**	54.70	1.65	0.05
**M3**	1.67	41.80	0.03	**M3**	380.60	19.40	0	**M3**	488.90	14.30	0	**M3**	51.40	0.74	0.04
